# Comparison of Aesthetic, Mechanical Outcome, and Bone Loss in Angulated Screw Channels (ASCs) and Cement-Retained Implant-Supported Prosthesis: A Case-Control Study

**DOI:** 10.3390/dj12080233

**Published:** 2024-07-24

**Authors:** Edoardo Rella, Paolo De Angelis, Laura Papetti, Giovanni Damis, Giulio Gasparini, Antonio D’Addona, Paolo Francesco Manicone

**Affiliations:** 1 Division of Oral Surgery and Implantology, Department of Head and Neck, Oral Surgery, and Implantology Unit, Institute of Clinical Dentistry, IRCSS “A. Gemelli” Foundation, Catholic University of the Sacred Heart, 00168 Rome, Italy; dr.paolodeangelis@gmail.com (P.D.A.); laura.papetti@unicatt.it (L.P.); giovanni.damis@unicatt.it (G.D.); antonio.daddona@unicatt.it (A.D.); paolofrancesco.manicone@unicatt.it (P.F.M.); 2 Maxillo-Facial Surgery Unit, IRCSS “A. Gemelli” Foundation, Catholic University of the Sacred Heart, 00168 Rome, Italy; giulio.gasparini@unicatt.it

**Keywords:** dental implant-abutment design, dental prosthesis design, dental implants

## Abstract

Angulated-screw channels (ASCs) allow the clinician to employ screw-retained restorations in almost all cases, as the access hole can be moved away from the vestibular portion of the crown, where it would jeopardize the final esthetic result. The objective of this study was to compare screw-retained restorations employing ASCs with restorations cemented on angled abutments. In this study, 30 subjects, equally divided into two groups: group 1 (cemented restorations on angulated abutments) and group 2 (screw-retained restorations adopting ASCs), were treated and retrospectively compared after 2 years using the pink esthetic score (PES) and the white esthetic score (WES). All restorations were in use at the last follow-up, with a survival rate of 100%. Three mechanical complications were observed (2 chippings and 1 crown came loose), with a success rate of 93% in group 1 and 87% in group 2 (*p* > 0.05). No statistically significant differences were reported regarding the esthetic outcome; the marginal bone loss (MBL) showed better results for the screw-retained restorations, both at the distal aspect (group 1 = 0.98 mm ± 0.16; group 2 = 0.45 mm ± 0.06; *p* = 0.006) and at the mesial aspect (group 1 = 1.04 ± 0.27; group 2 = 0.45 ± 0.005; *p* < 0.001). From an esthetical perspective, screw-retained restorations with ASCs and cemented restorations on angulated abutments are both effective means of restoring implants; both have excellent esthetic outcomes, but screw-retained restorations have reduced bone loss when compared to cemented ones but are more prone to mechanical complications. Still, our results must be cautiously observed given the reduced dimension of our sample. Larger studies are needed to confirm our findings.

## 1. Introduction

Implant-supported prosthetics are a successful option for rehabilitating partially or totally edentulous patients, given their efficacy in restoring an appropriate esthetic appearance and providing excellent functionality [1,2], as many papers have stated a success rate that goes over 95% [3].

These restorations can be connected to the implant in two main ways: through cement-retention or screw-retention mechanisms. While cementing implant restorations to an abutment screwed to an implant may seem straightforward, clinical evidence suggests a link between biological complications and excess cement in the peri-implant tissues [4]. Similarly, retrieving the prostheses if any complications arise can be difficult, especially when compared to the easier retrieval process that is needed for screw-retained restorations [5].

Moreover, the challenges of maintaining and retrieving cemented restorations have led to a progressive preference for screw-retention mechanisms. However, there are instances where implants are not ideally positioned for screw-retained restorations, which would involve placing the access hole on the facial surface of the crown, or in a position where it would jeopardize the veneering ceramic by reducing its thickness [6]. Moreover, cemented restorations can offer more reliably a true passive fit of the restoration, ensuring no further tension on the implants.

This issue often arises in the anterior maxilla due to bone resorption patterns and bone shape [7,8]. Additionally, angled implant placement in posterior areas may be necessary to avoid sensitive structures like the maxillary sinus or mandibular canal. In such cases, correcting the implant’s angulation might involve using an angled abutment with a cement-retained restoration or resorting to surgical bone augmentation to facilitate better implant placement. Alternatively, angle correction can be achieved through intermediate abutments or through restorations held in place by a lateral screw. Nevertheless, these methods are known to increase the complexity of treatment, maintenance requirements, and overall costs [9].

In the last decade, a design known as the angled screw channel (ASC) abutment (Dynamic Abutment; Talladium International Implantology, Valencia, Spain) was introduced to simplify the process of restoring angulated implants with directly attached, screw-retained restorations [10,11]. The ASC design employs a screw with a hexalobular head shape [12] that can be fastened with a hexagonal faceted sphere screwdriver at various angles ranging from 0 to 28 degrees, offering 360-degree rotational freedom [13,14]. This design enables the tightening of the abutment screw at an orientation different from the implant’s central axis [15]. Initially, ASC abutments were created on a hemisphere base using a burnout sleeve that could be freely rotated to direct the screw access channel away from problematic areas. Furthermore, this concept was initially limited to specific implant systems [16]. Recent advancements in implant software and manufacturing systems have made it possible to digitally design and fabricate ASC restorations [17,18]. Additionally, some implant manufacturers now offer prefabricated titanium bases that incorporate the ASC feature. The versatility of the ASC design has been validated by a cone-beam computed tomography (CBCT) analysis study, which demonstrated that screw-retained restorations can be achieved using ASC abutments in 76% of cases in the anterior maxilla [7]. Despite the growing popularity of this approach, the efficiency and long-term success of ASC systems remain uncertain, as few clinical reports have evaluated the efficacy of ASC in single crowns [17,19,20] but have shown promising results with a success rate of up to 95% after 4 years. The main drawbacks behind these restorations have been hinted at in the literature. First and foremost, the hexalobular head shape may not allow for adequate torque when screwing the crown to the implant; moreover, in order to allow a proper screwdriver engagement at the lingual aspect of the crown, the ASC system requires a screw channel widening for the required angulation design. This results in Zirconia thinning of some lingual and cervical areas, undermining the overall prosthesis strength [10].

Nothdurft and colleagues conducted a study comparing the strength of zirconia implant abutments with straight and angled designs [21]. They found that restorations using angled abutments had a higher average fracture resistance compared to those with straight abutments. Another investigation revealed that when the implant axis is tipped lingually, it significantly decreases the fracture strength of angle-corrected zirconia abutments [22]. Prior research also compared the fracture resistance of angulated screw channel (ASC) abutments with different screw angulations [23]. It showed that Zirconia abutments with a 25° angulated screw channel have notably lower fracture resistance than those with a straight channel. Consequently, to minimize mechanical issues, manufacturers have limited the recommended angles for zirconia abutments to 15° to 20° for stock abutments and 30° for custom abutments. However, despite these guidelines, incidents of zirconia abutment fractures still occur and are regularly documented [24].

Screw-retained restorations should therefore, at least on paper, provide several advantages: these restorations can be easily retrieved [25], there is no risk of having excess cement in the peri-implant tissues [26], and given the subgingival profile of angled abutments, which have a higher titanium neck, they should also offer a better esthetic [27]. Therefore, they should have a lower risk of bone loss, and given that the dental laboratory can better adapt the crown to the tissue profile, it is a better esthetic when compared to stock, angled abutments. On the other hand, cemented restorations could offer a better “passive fit” and do not have an access hole; on the other hand, retrieving this kind of restoration is quite hard and sometimes leads to the fracture of the prosthesis [24].

The objective of this study was to compare screw-retained restorations employing ASCs with restorations cemented on angled abutments when applied to implant-supported single crowns, comparing the MBL (marginal bone loss), the PES (pink esthetic score), and the WES (white esthetic score). The null hypothesis is that no difference, both in clinical and esthetic outcomes, will be found. 

## 2. Materials and Methods

Thirty patients that had previously received an implant-supported single crown between January 2020 and December 2020 and, given the implant/angulation, needed rehabilitation with a crown cemented on an angled abutment or with a screwed-retained prosthesis employing an angulated screw channel were included in the present study. Implants were from the same brand and were all bone-level implants. Given the retrospective nature of this article, ethical review and approval were waived by the ethical committee. This article was written following the STROBE guidelines for case-control studies (Supplementary Table S1).

We excluded patients based on the following exclusion criteria:

Inclusion

1. Patients that had received a single implant restoration cemented on stock abutments or screw-retained employing ASC.

Exclusion

1. Implant positioned in the posterior area (premolars to molars)
2. Restorations cemented on custom abutments.
3. Patients that had undergone previous radiotherapy to the facial district, suffered from diseases, or were under treatment with drugs that could impair bone metabolism. (i.e., bisphosphonates)

These patients were treated in different private practices by the same clinician, who has several years of experience in implant surgery. All implants were from the same manufacturer (Biomet 3i, Zimmer Biomet, Warsaw, IN, USA). All implants were placed after an appropriate healing time of 2 to 3 months; no implant-supported provisional restorations were provided for any of these patients. These patients were then divided into 2 groups according to the treatment they had received: Group CR (cement-retained) and Group SR (screw-retained).

These patients were followed up to 2 years after receiving the prosthesis. 

**Figure 1:**
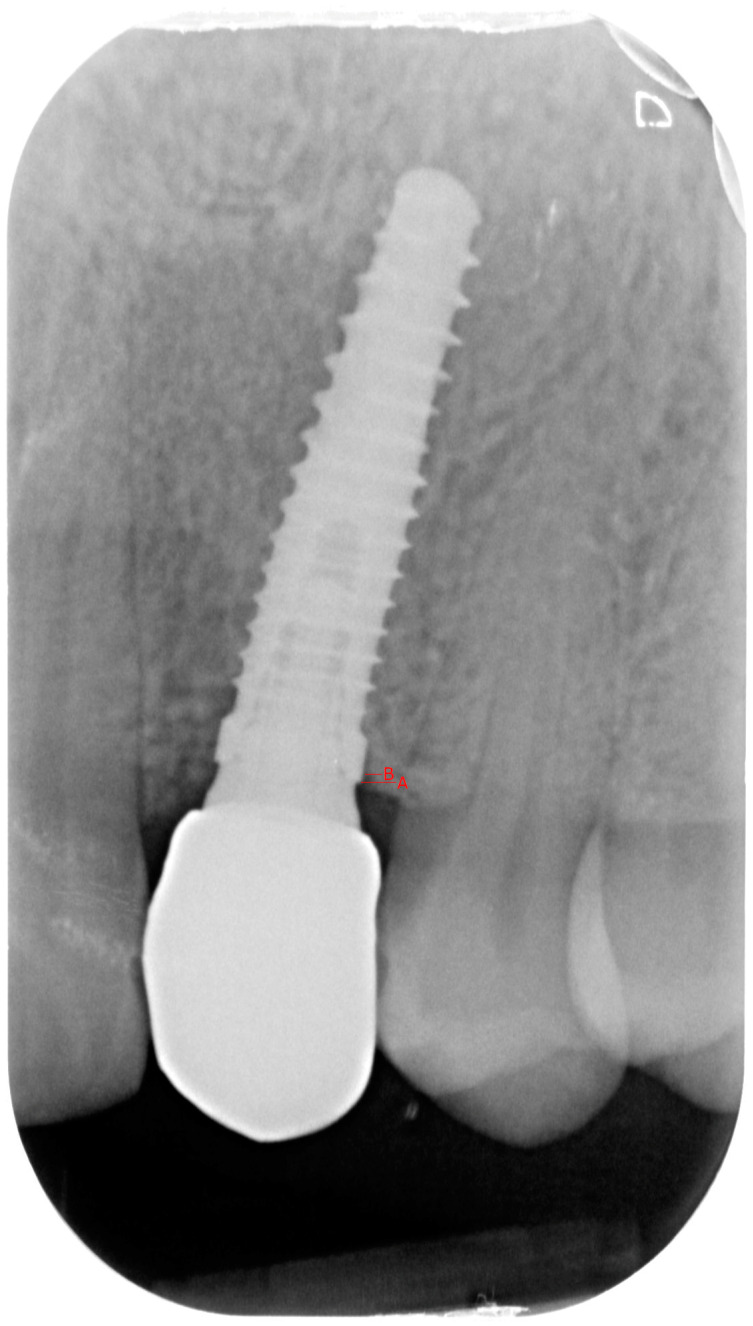
The linear distance between A (implant shoulder) and B (first point of bone to implant contact) was measured and compared between baseline and the last follow-up to measure the marginal bone loss.

**Figure 2:**
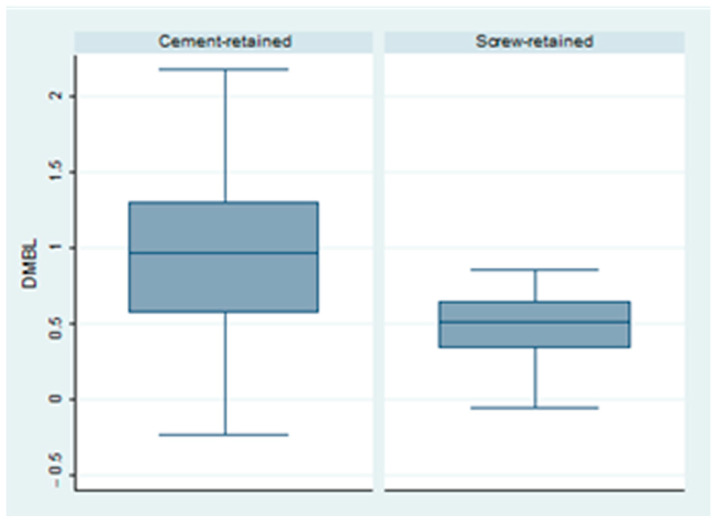
Box plot of the difference in distal bone level in both groups.

**Figure 3:**
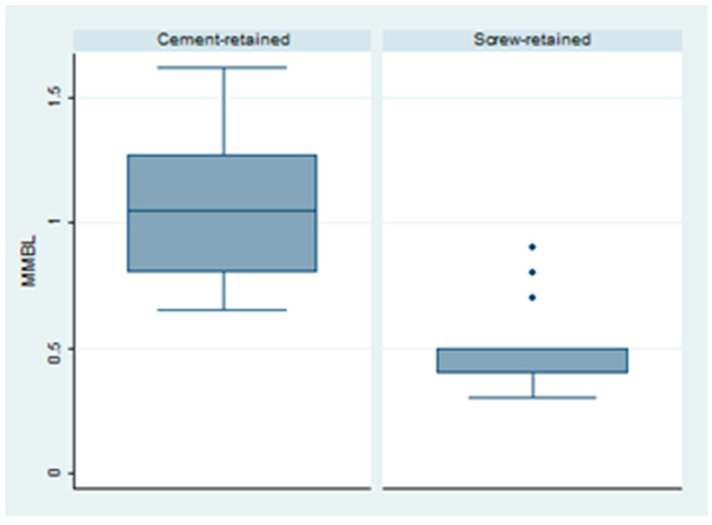
Box plot of the difference in mesial bone level in both groups.

**Figure 4:**
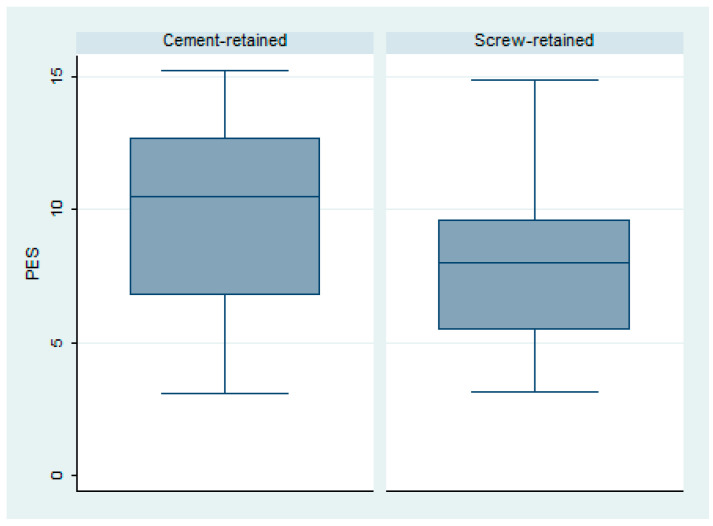
Box plot of the pink esthetic scale in both groups.

The treatment protocol that was followed is hereby explained:

1. Group 1: An implant-level impression adopting an open-tray impression technique was adopted using a polyvinylsiloxane impression material. After pouring the cast, an angled abutment was chosen, and a partially-veneered zirconia crown (limiting the veneering process to the vestibular surface) was obtained. At a second appointment, the abutment was screwed to the implant, and the crown was checked following the CMO (contact, margins, occlusion) acronym. The abutment was then manually screwed to the implant and secured at 25 Ncm. The access hole was closed with Teflon tape, and the crown was cemented with glass ionomer cement. (FujiCem 1, GC Europe, Leuven, Belgium) (Figure 1 and Figure 2).
2. Group 2: An implant-level impression adopting an open-tray impression technique was adopted using a polyvinylsiloxane impression material. After pouring the cast, a T-Base for ASCs was chosen, and a partially-veneered zirconia crown (limiting the veneering process to the vestibular surface) was realized. An angulated screw channel was designed, moving the access hole to the occlusal/lingual surface. At a second appointment, the crown was screwed to the implant and checked following the CMO (contact, margins, occlusion) acronym. The crown was then manually screwed to the implant and secured at 25 Ncm. The access hole was closed with Teflon tape and flowable composite resin. (Figure 3 and Figure 4).

These patients were then followed for 2 years after delivery.

At the last follow-up, the white esthetic score (WES) and pink esthetic score (PES) were measured by another clinician who did not take part in the treatment process [28].

1. PES: The PES comprises the following five variables: the mesial papilla, the distal papilla, the curvature of the facial mucosa, the level of the facial mucosa, and soft tissue color and texture at the facial aspect of the implant site. A score of 2, 1, or 0 is assigned to all five PES parameters. The two papillary ratings (one for the mesial and one for the distal) are used to evaluate the extent of papillary tissue present, with a full papilla receiving a score of 2, a partial papilla receiving a score of 1, and a complete absence receiving a score of 0. The contour of the facial soft tissue line, which is also known as the point where the implant restoration emerges from the soft tissues, is assessed for its similarity to a natural control tooth. It is rated as nearly identical (score 2), slightly different (score 1), or significantly different (score 0), determining whether it creates a natural and balanced appearance or a less harmonious one.

This index also combines three more specific soft tissue parameters into one variable, and a score is assigned based on the presence or absence of these parameters. The three parameters are the profile of the facial aspect, the color of the mucosa, and the texture of the gingival surface.

2. WES: The WES concentrates specifically on the visible section of the restoration, referring to the implant crown emerging from the peri-implant mucosa. This evaluation is based on five key parameters: the overall shape of the tooth, the contour and size of the clinical crown, color evaluation encompassing hue and value, surface texture, and translucency with characterization. Each of the five parameters is given a score of 2, 1, or 0. Therefore, in the case of an ideal implant restoration, a maximum WES score of 10 is attained. These parameters are evaluated by directly comparing them with the natural, opposing tooth, determining the degree of similarity or any possible differences. If the esthetic aspects of the reference tooth are optimally replicated, a perfect WES score of 10 can be achieved. The accepted clinical threshold remains at a score of 6.

Marginal bone loss (MBL) was calculated according to the following protocol, further explained in Figure 1: a radiograph was taken immediately after having delivered the crown, and another one was taken at the last (2-year) follow-up. 

The radiographic linear distance from the implant shoulder to the first bone-to-implant contact was used to calculate the marginal bone levels. All the radiographs were taken by applying the long cone technique and by using a film holder. All radiographs were taken using the same X-ray equipment (CS2100, Carestream, New York, NY, USA) and the same Phosphor Sensor brand (Vistascan, Durr Dental, Bietigheim-Bissingen, Germany). To consider the anatomic magnification and distortion in the films, the linear dimensions of the digitized images were calibrated. This was achieved by setting the scale in the image to the known distance between two implant threads. The radiographic bone loss was calculated by subtracting the marginal bone level at baseline from the marginal bone level at the follow-up examination. The values were rounded to 1/10 mm. The same operator was in charge of measuring these values, and the operator was calibrated on the same 30 images (Cohen’s kappa = 0.96).

### Statistical Analysis

Categorical variables were presented as absolute and relative frequency, while numerical variables were presented as mean ± standard deviation, and/or as median and min/max range. Normality was checked with the Shapiro–Wilk test and by visually observing the distribution. In order to compare the recorded outcomes between Group 1 and Group 2, the Student’s *t*-test was used for normally distributed variables.

A post-hoc analysis was realized, adopting the marginal bone level as the selected variable and measuring a statistical power of 0.34.

## 3. Results

Thirty patients were recruited for the present study, with a mean age of 44.18 ± 7.67. 18 patients were men, and 12 were women.

Subjects were equally distributed among the two groups. No implants were lost in the follow-up, defining a 100% implant survival rate in both groups. Similarly, all prostheses were still in use at the last follow-up, defining a 100% prosthesis survival rate in both groups.

Three complications were observed: two crowns in Group 2 showed a minor chipping of the veneering ceramic, which was easily corrected by polishing and reshaping the ceramic; a crown in Group 1 came loose and had to be cemented. Overall, a 90% success rate was observed, with a 93% success rate in Group 1 and an 86% success rate in Group 2 (*p* > 0.05).

An overall distal bone loss of 0.715 mm ± 0.54 was observed at the last follow-up. Group 1 showed a mean DBL of 0.98 mm ± 0.16, while Group 2 showed a mean DBL of 0.45 mm ± 0.06. This difference was deemed to be statistically significant (*p* = 0.006) (Figure 2).

A mesial bone loss of 0.75 ± 0.36 was observed at the last follow-up. Group 2 (0.45 ± 0.005) had a lower value when compared to Group 1 (1.04 ± 0.27); this difference was deemed to be statistically significant (*p* < 0.001) (Figure 3).

The pink esthetic scale (PES) showed an overall mean value of 8.79 ± 3.57; Group 1 had an overall better esthetic result, with a mean value of 9.54 ± 0.99, while Group 2 had a mean value of 8.04 ± 0.84. This difference was not statistically significant (*p* = 0.25) (Figure 4).

**Figure 5:**
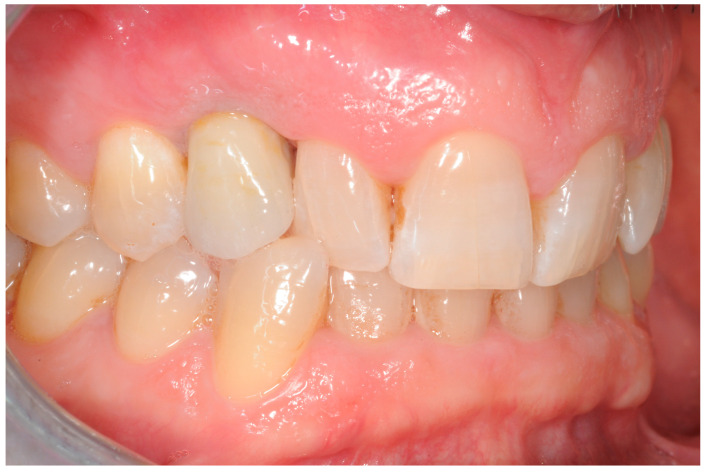
Implant-supported crown replacing tooth 1.3. The crown is cemented on the implant on an angulated abutment.

**Figure 6:**
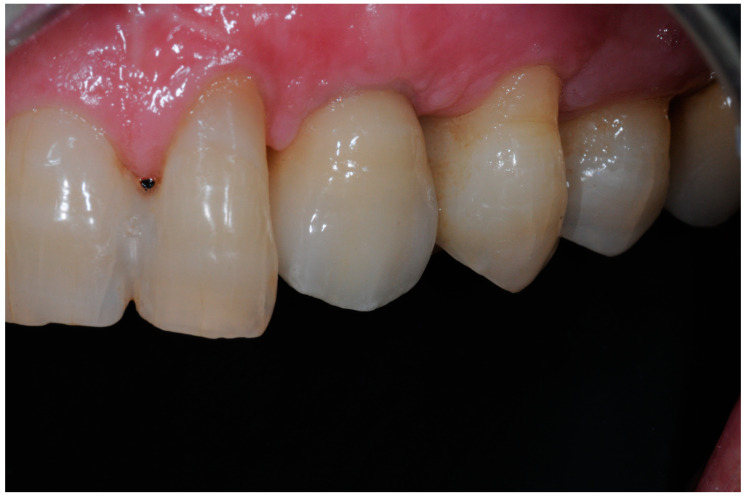
Implant-supported crown replacing tooth 2.3. The crown is screwed on the implant employing the angulated screw channel (ASC) technology.

**Figure 7:**
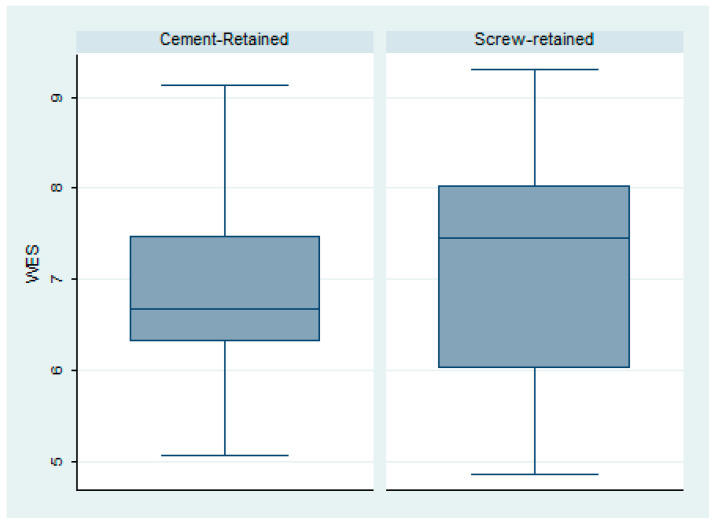
Box plot of the white esthetic scale in both groups.

The white esthetic scale (WES) overall results were 7.07 ± 1.18; Group 2 (Figure 5) had a better result regarding the esthetic of the crown, with a mean value of 7.18 ± 0.36, while Group 1 had a WES of 6.96 ± 0.26 (Figure 6). This difference was not statistically significant (*p* = 0.62) (Figure 7).

## 4. Discussion

The present case-control study compares esthetic and biologic outcomes between screw-retained, implant-supported single crowns employing an angulated screw channel and classical cemented-retained, implant-supported single crowns in the anterior region.

The results hint that an immediate favorable esthetic result can be obtained using both techniques; what matters most is the correct implant positioning and good treatment of the soft tissues. 

The adoption of angulated screw channels has theoretically a few advantages. First of all, moving the access channel from the vestibular surface to the occlusal one can improve the esthetical outcome of the final prosthesis, as closing the access hole in composite can be esthetically unappealing.

This allows the clinician to adopt a screw-retained restoration in place of cemented or conically retained ones. While both kinds of rehabilitations have a good amount of literature in their support and can provide good results even after long follow-ups, they both have their inherent disadvantages: Cemented-retained crowns have the well-documented risk of leaving traces of cement in the peri-implant sulcus, which could very well lead to peri-implantitis, jeopardizing the health of the implant; moreover, they are much more difficult to retrieve and remove if any complications emerge. On the other side, as no access hole is present, their esthetic could be defined as marginally better, and they can be adopted thanks to angulated abutments and custom abutments in any clinical situation. Conometric-retained restorations, on the other hand, have both no risk of leaving cement in the peri-implant sulcus and no access hole, but not all implant manufacturers support them, and long-span bridges can be complicated to manufacture [29]. 

Our paper shows no esthetically significant difference between the two major forms of retention, as both cemented retained and crew retained restorations had more than average results. Similarly, no difference was measurable in terms of success or survival rate. This might indicate, as stated in many other papers, that both kinds of restorations can be adopted and might be an appropriate solution for most cases [2,3]. Our results are only relative to the first couple of years of a restoration. In order to confirm that screw-retained restorations employing ASCs can offer similar esthetic results to those obtained with cemented restorations, more studies with longer follow-ups are needed.

Our results, even if only a small sample was investigated, are also in line with what other papers have found: that screw-retained restorations are exposed to a higher risk of mechanical complications, such as chipping of the veneering ceramic, as many other papers have found [30]. We did not observe an augmented risk of screw-loosening with angulated screw channels, which was postulated given the reduced preload that can be applied to the screw [31,32]. This observation is in line with what a recent systematic review has found [33]. Similarly, no cases of screw fracture were observed, showing that, similarly to what other reviews have found, these restorations do not have an increased risk of this type of complication [34]. Our short follow-ups can only allow us to relate these findings to the first couple of years of an implant-retained restoration; in order to further advance our findings, therefore, longer follow-ups are needed.

With regards to the radiographically measured bone loss, screw-retained restorations had a lower value than cemented restorations, and this difference was found to be statistically significant. This can be associated with the inherent advantages of screw-retained restorations, which do not employ cement and therefore have a lower risk of causing inflammation of the supporting tissues [35]. This is similar to what other studies found: screw-retained restorations, given their retention method, tend to provoke less tissue inflammation, which could therefore reduce the amount of bone loss or bleeding from probing [36]. Some studies have also linked ASCs to a reduction in the concentration of pro-inflammatory cytokines, which corroborates our clinical findings given the role these extracellular mediators play in bone metabolism [37]. These differences are statistically significant and may be clinically relevant; our study has a quite short follow-up (only 2 years), but even after such a short follow-up, a difference of 0.50 mm over two years might, at longer follow-ups, account for a significant bone loss that might even lead to the loss of the implant or an unaesthetically pleasing restoration with exposure to the implant fixture. Further studies with longer follow-ups are needed to confirm this observation. It is completely possible that the bone resorption that we have found is only part of the bone loss that frequently manifests after exposing and loading the implant, in which case the marginal bone loss of the two different retention methods might, at longer follow-up, eventually converge. 

Our paper has many limitations; its case-control nature and the therefore non-random allocation of patients to the treatment groups is a major bias as many other factors could have had an effect on the reported variables; moreover, patients were only followed for a brief period of time after their treatment; therefore, no inferences can be made on longer time spans (after 10 or 20 years). Longer studies are therefore needed to confirm our findings, but, given the relative novelty of ASC, we believe our findings are an important stepping stone in building our knowledge. Stock-angled abutments were adopted, while no information is provided regarding the performance of custom milled (either metal- or zirconia-based) abutments, which could theoretically further improve the results obtained with cemented restorations. The MBL was also measured only on bidimensional radiographs, so no information is available regarding the bone loss on the vestibular and lingual surfaces. Another shortcoming of our paper is the reduced sample size, as only 30 subjects were included. Furthermore, the clinician in charge of measuring the PES and WES in this article was not calibrated, and, given the design of this study, patients were not randomly allotted to one treatment rather than the other. 

## 5. Conclusions

Cement-retained restorations and screw-retained restorations are two valuable alternatives when rehabilitating single implants, as both are capable of providing a stable, esthetically pleasing result up to 2 years after delivery. Our results hint that screw-retained restorations employing angulated screw channels might be more stable in terms of bone stability while providing the same esthetical and functional result; more studies are needed to confirm this observation, given the small sample we have observed in our paper.

## Data Availability

Data are available on request to the corresponding author.

## Supplementary Materials

The following supporting information can be downloaded at: https://www.mdpi.com/article/10.3390/dj12080233/s1, Table S1: STROBE Statement—Checklist of items that should be included in reports of case-control studies.
